# Analyzing the impact of human leukocyte antigen mismatch on the incidence of prostate cancer and the advantage of T cell therapy in patients after kidney transplantation based on the United Network for Organ Sharing database

**DOI:** 10.3389/fonc.2025.1562869

**Published:** 2025-09-10

**Authors:** Yang Gao, Jingjing Li, Jiaxi Mao, Aijun Jiang, Wenyuan Guo, Shangxi Fu

**Affiliations:** ^1^ Department of Hepatobiliary Surgery, People’s Liberation Army of China (PLA) Navy Characteristic Medical Center, Shanghai, China; ^2^ Department of Organ Transplantation, Shanghai ChangZheng Hospital, Shanghai, China; ^3^ Department of Urology, Kidney Transplantation Center, Ruijin Hospital, Shanghai Jiaotong University School of Medicine, Shanghai, China

**Keywords:** kidney transplantation (KT), HLA typing, prostate cancer, UNOS/OPTN, induction therapy

## Abstract

**Background:**

We aim to analysis the impact of Human Leukocyte Antigen (HLA) mismatch between kidney transplant donors and recipients on the incidence of prostate cancer after kidney transplantation (KT). Meanwhile, understanding the use of T cell therapy is of great importance after kidney transplantation from the perspective of prostate cancer occurrence.

**Methods:**

A retrospective study was conducted on kidney transplant recipients based on the United Network for Organ Sharing (UNOS) database from 2000 to 2019. General demographic data, socio-economic and educational data, personal medical history, immunosuppressive therapy regimens, and HLA typing of donors and recipients were collected to analyze the impact of: (1) baseline patient characteristics; (2) HLA mismatch; and (3) HLA subtype mismatch on the incidence of prostate cancer after transplantation.

**Results:**

A total of 268–994 kidney transplant recipients were included, with 1–910 newly diagnosed prostate cancer patients after surgery. Both univariate and Cox multivariate analysis discovered that the use of T cell therapy could reduce the risk of prostate cancer after KT [0.89(0.86~0.91)]. We also found HLA mismatch ≥ 3 is a risk factor of prostate cancer after transplantation [1.07(1.02~1.11)]. Further subgroup analysis was conducted on HLA mismatch. The Cox multivariate analysis of HLA-A (0–2), HLA-B (0–2), and HLA-DR (0–2) mismatch showed that 2-mismatch in HLA-A and HLA-B was a risk factor of prostate cancer after KT [1.19(1.01~1.40)]; 2-mismatch and 1-mismatch were both risk factors of prostate cancer after KT in the HLA-DR group [1.32(1.13~1.54)], [1.20(1.03~1.39)].

**Conclusions:**

From the perspective of prostate cancer occurrence after transplantation, the use of T cell therapy is of great significance. HLA mismatch ≥ 3 was a risk factor of prostate cancer after KT. HLA-A and HLA-B 2-mismatch were risk factors of prostate cancer after KT, while HLA-DR 1-mismatch and 2-mismatch were both risk factors of prostate cancer after KT. This research contributed to the focus on the relationship between induction therapy and cancer occurrence after KT, and also provide guidance for reasonable selections of HLA typing of prostate cancer before KT.

## 1 Introduction

Kidney transplantation commonly represents the optimal treatment for patients with end-stage renal disease (ESRD) (1–7). Besides the undisputable advantages of transplantation, an increased incidence of malignancies after transplantation was shown (8, 9). Malignant tumor is the second leading cause of death for kidney transplant recipients (10). Compared with non-transplant population, the incidence rate of malignant tumor in patients after kidney or liver transplantation is 2–4 times higher (11, 12). Common malignant tumors after KT include skin cancer, post-transplant lymphoproliferative disease, and urologic tumors (13). Worldwide, prostate cancer is the second most common form of solid tumor in men, surpassed only by nonmelanoma skin cancers such as basal and squamous cell carcinomas. In this research, we choose prostate cancer to analysis the impact of HLA mismatch after KT (14, 15).

Malignancies have been identified to be widely associated with multiple baseline patient characteristics, such as age, gender, race, smoking, body mass index (BMI), ABO blood types, education background, duration of dialysis, etc. (16–19). In addition, the polymorphism and expression of Human leukocyte antigen (HLA) molecules have also been demonstrated to be correlated with the occurrence and development of tumors (20). HLA is the expression product of human histocompatibility complex (MHC) (21). HLA is categorized into class I, II, and III (22–25). The HLA class I molecules include HLA-A, HLA-B, and HLA-C; the HLA class II antigens include HLA-DP, HLA-DQ, HLA-DR; and the HLA class III antigens encode complements, cytokines, and heat shock proteins (26–32). HLAs play an essential role in the cellular and humoral immune responses after KT, as well as in determining the outcome of the transplants (33). It is widely recognized that the fewer mismatches of HLA-A, HLA-B, and HLA-DR between donors and recipients in KT, the smaller the rejection reaction, and the higher survival rate of the transplanted kidney (21, 34, 35). However, finding a well-matched donor may not be possible for all patients and usually prolongs waiting time (36). Furthermore, the introduction of modern immunosuppressive regimens markedly declined the emphasis on HLA matching (37). Even though using immunosuppressants after transplantation has been proved to improve the survival rate of both the recipients and the transplanted kidneys (34, 38–42), it can still increase the risk of infection and malignancy, as well as other attendant side effects (36). Therefore, understanding the effects of HLA mismatching on recipients is of great importance for increasing graft survival and further reducing the risk of recipient mortality. Previous studies on HLA mismatch have mainly focused on acute rejection and kidney transplant prognosis after transplantation. However, how does HLA mismatch affect the incidence of tumor after KT remains unclear. To this regard, our study aims to bridge this gap by analyzing the impact of both HLA mismatch and HLA subtype mismatch on the incidence of prostate cancer after KT in the United States. In addition, we also explore the necessity of induction therapy after KT from the perspective of prostate cancer occurrence. In order to make this retrospective study more comprehensive, the role of multiple baseline characteristics of the transplant recipients in the incidence of prostate cancer after KT were also investigated. We expect this retrospective study can provide the reference for prostate cancer screening and treatment strategies after KT.

## 2 Materials and methods

### 2.1 Study population

**Figure 1:**
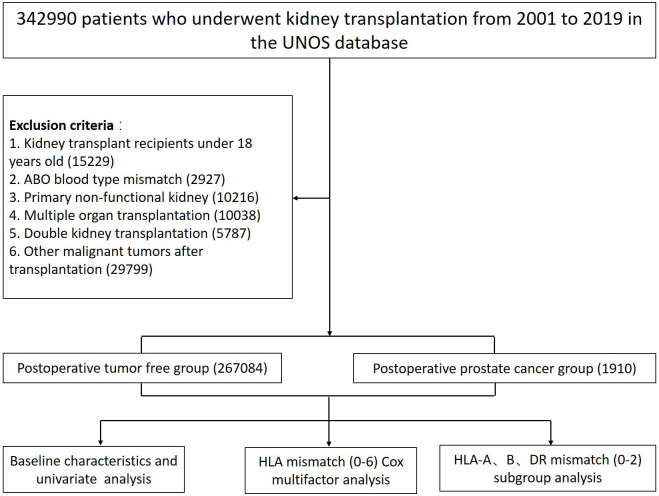
Flow diagram of patients who underwent KT from 2000 to 2019 of the UNOS database.

The present study retrospectively investigated all adult kidney transplant patients (i.e., age at transplantation ≥ 18 years) from 1 January 2000 to 31 December 2019 of the United Network for Organ Sharing (UNOS) database. Recipients with the following conditions including: (1) ABO blood type mismatch; (2) primary non-functional kidney; (3) multiple organ transplantation; (4) double KT; and (5) recipients developing other malignant cancers other than prostate cancer were excluded. 267–084 patients matched the inclusion criteria of whom 1–910 developed prostate cancer after KT were ultimately included (**Figure 1**).

### 2.2 Covariates and outcomes

The UNOS database records data regarding both recipient and allograft characteristics for all patients who received transplants. 14 variables were extracted from the UNOS database, including age, race, BMI, ABO blood type, cause of ESRD, commercial insurance, education level, history of malignant tumor, acute rejection, duration of dialysis, transplantation type, HLA mismatch number, whether to use interleukin-2 receptor subunit alpha (IL-2RA) or T cell therapy, and the type of immunosuppressive agents being used (i.e., cyclosporine (CSA), tacrolimus (TAC), mycophenolic acid (MPA), and mammalian target of rapamycin (mTOR) inhibitors). T cell therapy included ATGAM, Thymoglobulin, and Alemtuzumab.

The main observed outcomes of the study include 5-year and 10-year patient survival rates, as well as survival rate of transplanted kidneys. Patient survival refers to the time from transplantation to the death of the recipient. Graft survival refers to the time from transplantation to the failure of the transplanted kidney. When calculating survival rate, delete patients who have been lost to follow-up or died. Patients were divided into postoperative tumor free group and postoperative prostate cancer group.

### 2.3 Statistical analysis

The continuous variables were summarized using means and standard deviations, and were expressed as percentage as well. The age was expressed as the median (interquartile range (IQR)). Chi-square test and t-test were used to compare the baseline feature classification variables and continuous variables of patients, respectively. Kaplan Meier survival curves were presented after KT. The influencing factors of prostate cancer incidence were analyzed using Cox multiple regression, and HR and 95% confidence interval (CI) were calculated. A p-value of <0.05 was considered significant for all analyses. Inspection level α=0.05 (double tailed). Analyses were performed using R 3.6.2.

Patients were divided into postoperative tumor free group and postoperative prostate cancer group based on whether malignant tumors occur after surgery. Cox multivariate analysis was conducted on HLA (0–6), HLA-A (0–2), HLA-B (0–2), and HLA-DR (0–2) mismatch.

## 3 Results

### 3.1 Baseline characteristics and univariate analysis of prostate cancer incidence after KT

Univariate analysis showed that gender, age, race, cause of ESRD, education level, commercial insurance, history of malignant tumor, living transplantation, HLA mismatch, and use of immunosuppressants were the significant influencing factors of postoperative prostate cancer. As compared to the tumor free group, the prostate cancer group had an older average age (59 vs. 51), more white recipients (52.2% vs. 49.0%), shorter dialysis duration before transplantation (2 vs. 3), more likely to get college or graduate degree (51.0% vs. 46.3%), more likely to have private insurance (42.0% vs. 36.3%), more likely to have history of malignant tumor (1.0% vs. 0.2%), less likely to have acute rejection (2.4% vs. 9.7%), more likely to have HLA mismatch ≥3 (82.5% vs. 79.3%); more likely to use interleukin-2 receptor subunit alpha (31.5% vs. 25.7%), more likely to use CSA, TAC, MPA, MTOR (13.0% vs. 10.6%), (69.6% vs. 61.9%), (80.7% vs. 73.5%), (7.1% vs. 5.7%), less likely to use T cell therapy (51.7% vs. 58.8%). The leading cause of ESRD in the prostate cancer group was hypertensive nephrosclerosis (27.2%), while in the tumor free group was diabetes mellitus (25.2%) (all P < 0.05) (**Table 1**). In this research, recipient ABO blood types and living transplantation have no significant difference between the tumor free group and prostate cancer group.

**Table 1:** Analysis of influencing factors for the incidence of prostate cancer in 268–994 recipients after KT.

Characteristics	No cancer	Prostate	P value
n	267,084	1,910	
Age (median [IQR])	51.00 [40.00, 60.00]	59.00 [53.00, 65.00]	<0.001
Recipient race (%)			<0.001
White	130915 (49.0)	998 (52.2)	
African American	71734 (26.8)	664 (34.8)	
Hispanic	43813 (16.4)	183 (9.6)	
Asian	15908 (6.0)	53 (2.8)	
Other	4714 (1.8)	12 (0.6)	
BMI ≥28 (%)	119026 (44.6)	864 (45.2)	0.572
Cause of ESRD (%)			<0.001
Glomerular diseases	49749 (18.6)	354 (18.5)	
Hypertensive nephrosclerosis	59268 (22.2)	520 (27.2)	
Polycystic kidneys	22974 (8.6)	235 (12.3)	
Diabetes mellitus	67230 (25.2)	432 (22.6)	
Retransplant	20603 (7.7)	95 (5.0)	
Other	47260 (17.7)	274 (14.4)	
Recipient ABO (%)			0.227
A	98669 (37.0)	745 (39.0)	
B	34551 (12.9)	249 (13.0)	
AB	13060 (4.9)	95 (5.0)	
O	120804 (45.2)	821 (43.0)	
Dialysis duration (median [IQR])	3.00 [1.00, 5.00]	2.00 [1.00, 4.00]	<0.001
Recipient education level (%)			<0.001
High school/GED or lower	116848 (43.8)	701 (36.7)	
College or graduate degree	123788 (46.3)	974 (51.0)	
Unknown	26448 (9.9)	235 (12.3)	
Private insurance (%)			<0.001
Yes	96852 (36.3)	803 (42.0)	
No	170232 (63.7)	1107 (58.0)	
History of malignant tumor (%)	602 (0.2)	19 (1.0)	<0.001
Living transplantation (%)	97352 (36.5)	721 (37.7)	0.25
Acute rejection (%)	25888 (9.7)	45 (2.4)	<0.001
HLA mismatch (%)			0.003
<3	54441 (20.4)	330 (17.3)	
≥3	211770 (79.3)	1575 (82.5)	
Unknown	873 (0.3)	5 (0.2)	
immunosuppression induction			
Interleukin-2 receptor subunit alpha (%)	68526 (25.7)	601 (31.5)	<0.001
T cell therapy (%)	156955 (58.8)	988 (51.7)	<0.001
immunosuppression maintenance			
CSA (%)	28246 (10.6)	249 (13.0)	0.001
TAC (%)	165338 (61.9)	1330 (69.6)	<0.001
MPA (%)	196240 (73.5)	1541 (80.7)	<0.001
MTOR (%)	15333 (5.7)	135 (7.1)	0.015

BMI, body mass index; ESRD, end-stage renal disease; GED, general educational development; HLA, human leukocyte antigen; CSA, Cyclosporin; TAC, Tacrolimus; MPA, Mycophenolic Acid; MTOR, mammalian target of rapamycin.

### 3.2 Survival analysis between tumor free group and prostate cancer group

**Figure 2:**
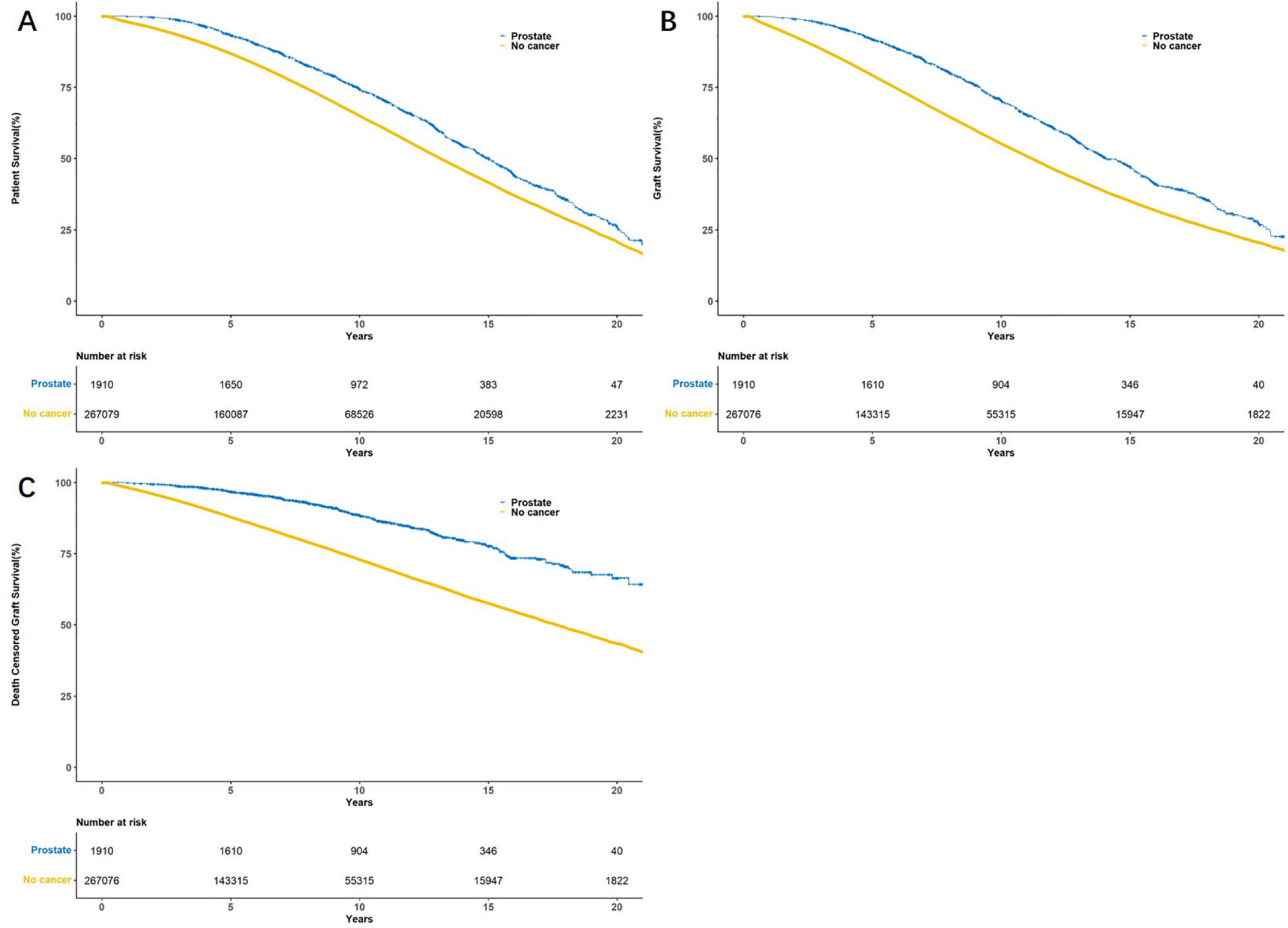
Kaplan–Meier survival curves showed patient survival **(A)**, graft survival **(B)** and death censored graft survival **(C)** between tumor free group and prostate cancer group.

**Figure 3:**
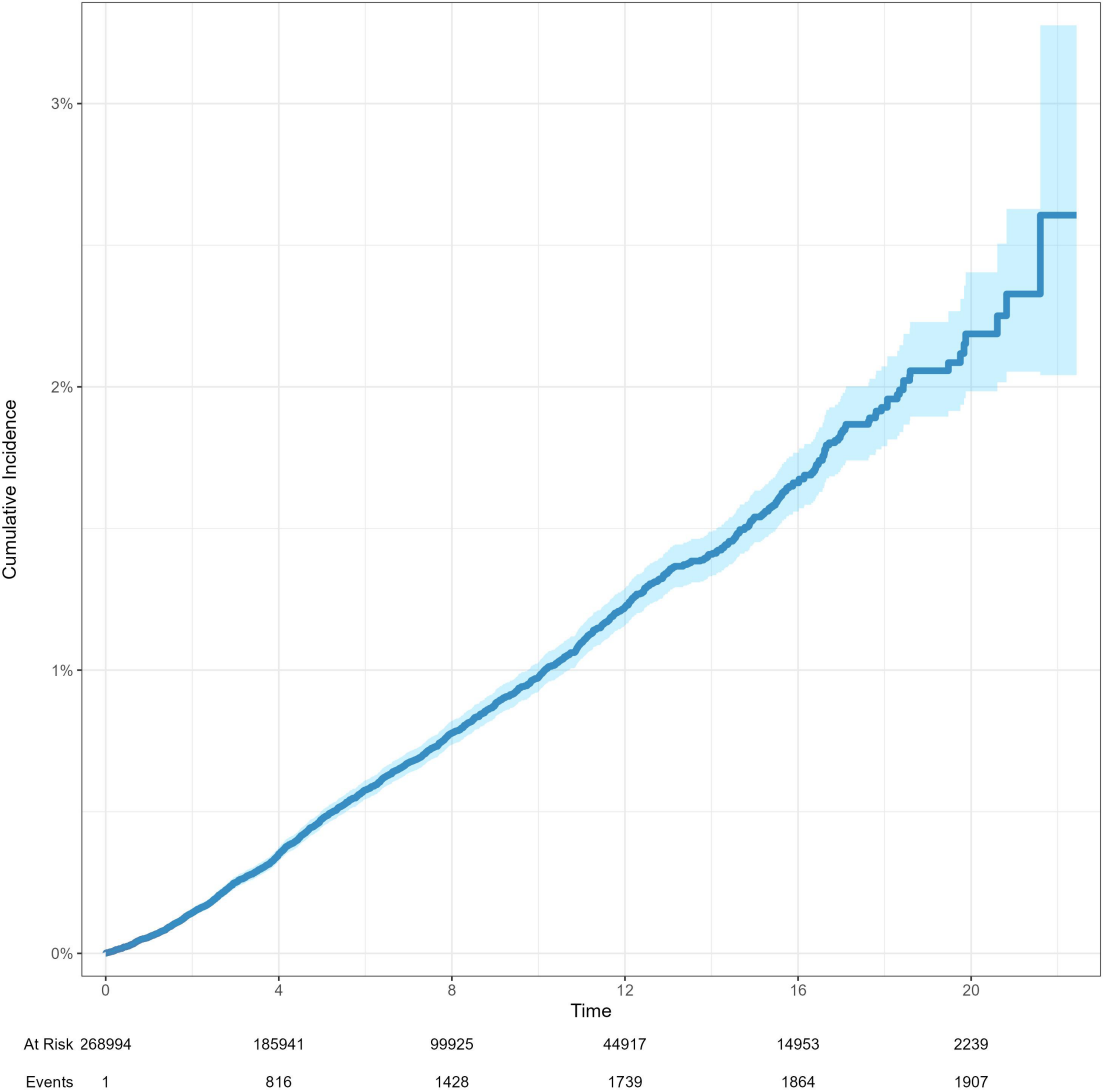
The cumulative incidence rates of prostate cancer after KT.

The 5-year and 10-year patient survival rates of postoperative tumor free group were 87.0% and 65.2%, respectively. The 5-year and 10-year patient survival rates of postoperative prostate cancer group were 93.4% and 74.4%, respectively. The 5-year and 10-year graft survival rates of postoperative tumor free group were 79.4% and 55.4%, respectively. The 5-year and 10-year graft survival rates of postoperative prostate cancer group were 91.9% and 70.4%, respectively. The 5-year and 10-year death censored graft survival rates of postoperative tumor free group were 88.0% and 73.1%, respectively. The 5-year and 10-year death censored graft survival rates of postoperative prostate cancer group were 96.8% and 88.5%, respectively (**Figure 2**). The cumulative incidence rates of prostate cancer and renal cancer after KT are presented in **Figure 3** and **Supplementary Figure 1**, respectively.

### 3.3 Cox multiple regression analysis of the influencing factors of prostate cancer incidence after KT

**Figure 4:**
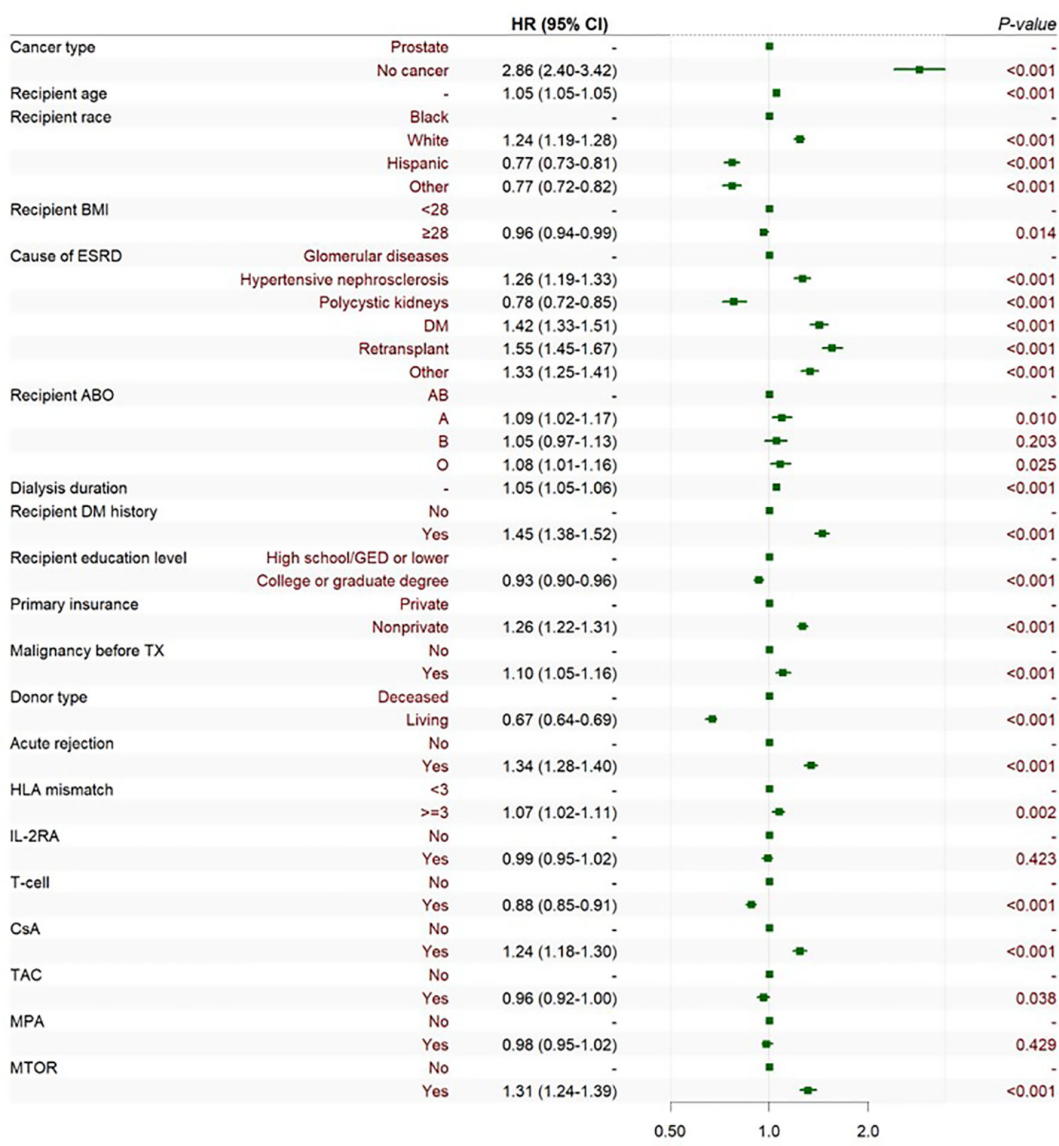
Cox multiple regression analysis of the influencing factors of prostate cancer incidence after KT. BMI is short for body mass index; ESRD is short for end-stage renal disease; DM is short for diabetes mellitus; GED is short for general educational development; TX is short for transplantation; HLA is short for human leukocyte antigen; CSA is short for Cyclosporin; TAC is short for Tacrolimus; MPA is short for Mycophenolic Acid; and MTOR is short for mammalian target of rapamycin.

**Figure 5:**
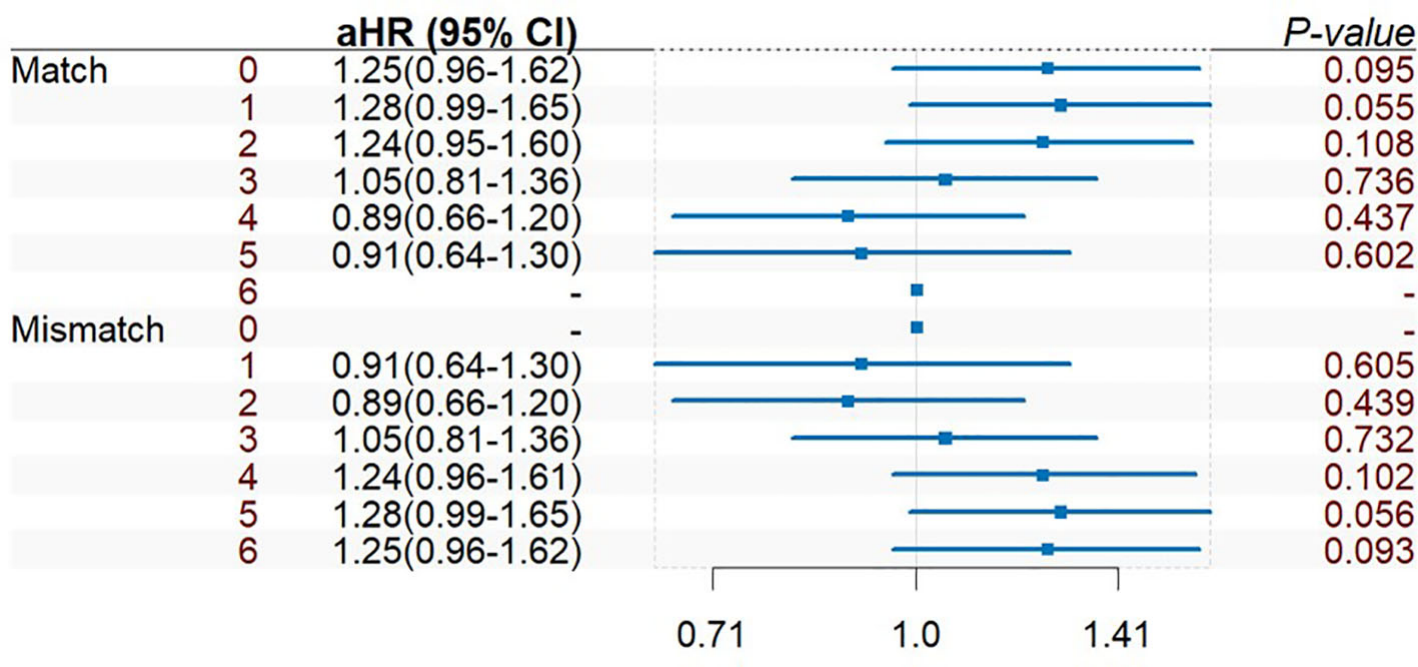
Multiple factor analysis of HLA match 0–6 and mismatch 0–6 of prostate cancer incidence after KT.

The adjusted Cox multivariate analysis showed that the elderly, white, history of malignant tumor, high education level, ESRD caused by polycystic kidney disease, nonprivate insurance, living transplantation, HLA mismatch ≥ 3, and the use of immunosuppressants were related to the incidence of prostate cancer after KT (**Figure 4**). HLA mismatch ≥ 3 is a risk factor of the occurrence of prostate cancer after KT [1.07(1.02~1.11)]. Multiple factor analysis was conducted on HLA match 0–6 and mismatch 0–6, while the results were not statistically significant (**Figure 5**).

**Figure 6:**
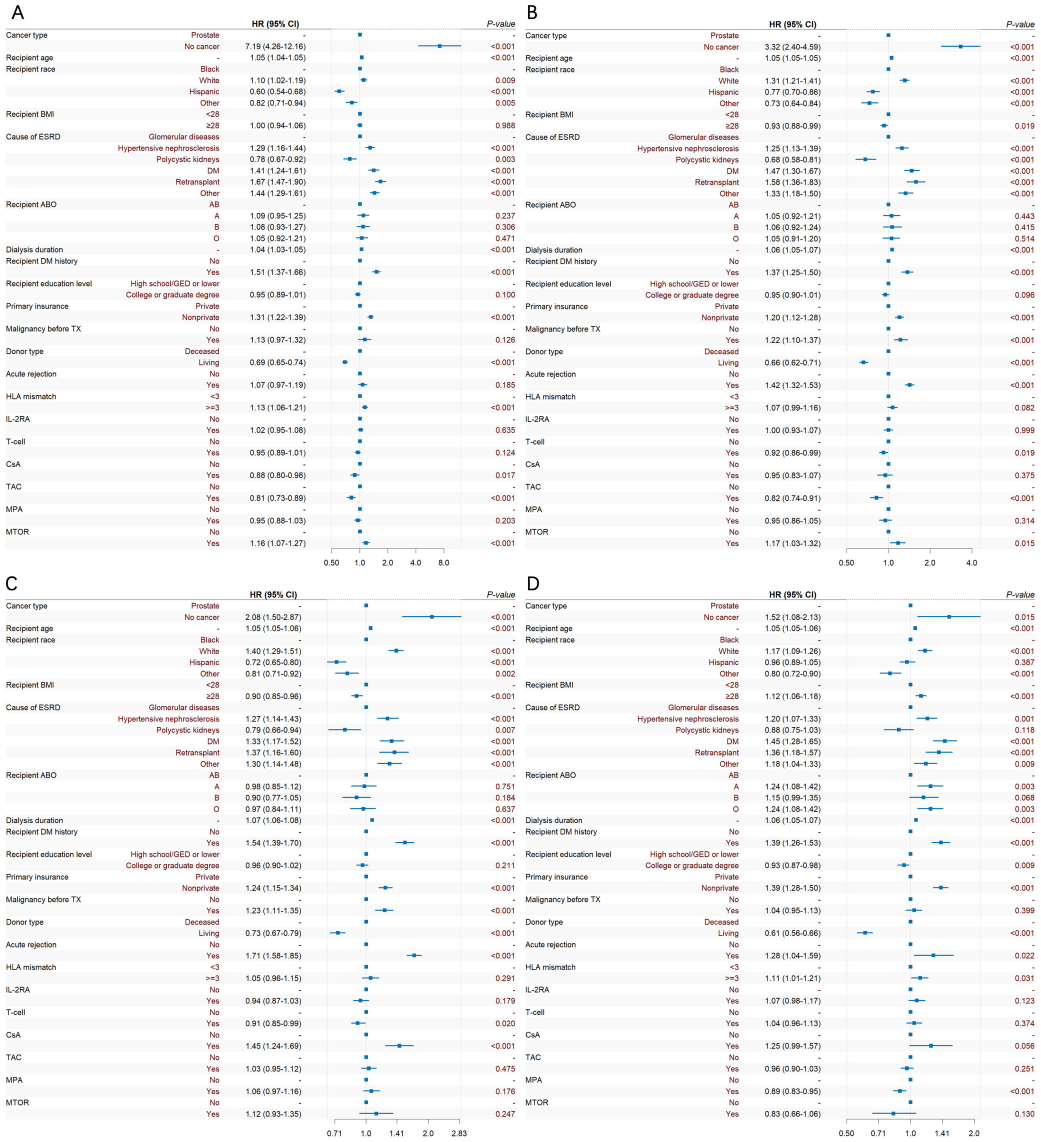
Temporal trends in prostate cancer risk factors after KT: Forest plots of Cox regression analyses stratified by era. **(A)** 2000-2004, **(B)** 2005-2009, **(C)** 2010-2014, **(D)** 2015-2019.

During induction therapy after KT, the use of T cell therapy could reduce the risk of prostate cancer [0.89(0.86~0.91)]. For maintenance therapy after KT, the use of TAC might be the protective factor of prostate cancer [0.92(0.96~1.00)], while CSA and mTOR could increase the risk of prostate cancer [1.24(1.18~1.31)], [1.31(1.24~1.39)]. Additional era-stratified analyses were performed to further validate our findings (**Figure 6**). During 2005–2009 and 2010-2014, T cell therapy was associated with reduced prostate cancer risk ([0.92(0.86~0.99)], [0.91(0.85~0.99)], respectively). This protective effect, however, did not reach statistical significance in the 2000–2004 and 2015–2019 cohorts.

### 3.4 Subgroup analysis of HLA match and mismatch on the occurrence of prostate cancer after KT

**Figure 7:**
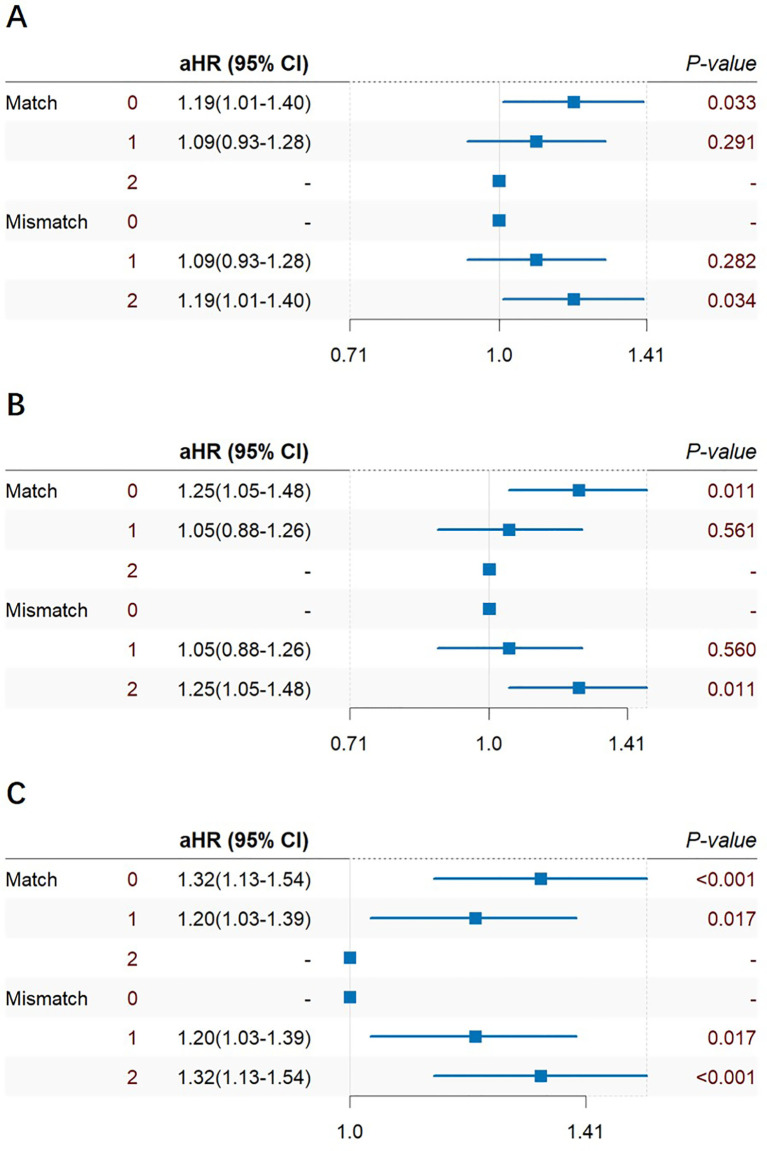
Cox multiple regression analysis of HLA-A **(A)**, HLA-B **(B)**, HLA-DR **(C)** match 0–2 and mismatch 0–2 of prostate cancer incidence after KT.

Cox multiple regression analysis was also performed on HLA-A, HLA-B, HLA-DR mismatch 0–2 and match 0–2 (**Figure 7**). In the subgroup analysis results, it was found that HLA-A, HLA-B, and HLA-DR groups had consistent HLA 0-match and 2-mismatch results, while HLA 1-match and 1-mismatch results were consistent. 2-mismatch in HLA-A and HLA-B groups was a risk factor of prostate cancer after KT [1.19(1.01~1.40)]; 2-mismatch and 1-mismatch were both risk factors of prostate cancer after KT in the HLA-DR group [1.32(1.13~1.54)], [1.20(1.03~1.39)].

## 4 Discussion

The Cox multivariate analysis showed that the elderly, white, history of malignant tumor, nonprivate insurance, HLA mismatch ≥ 3, and the use of CSA could all significantly increase the risk of prostate cancer after KT, while higher education, ESRD caused by polycystic kidney, living transplantation, the use of T cell therapy and TAC could reduce the risk of prostate cancer after KT. Additional era-stratified analyses were performed to clarify the protective effect of T cell therapy. Collectively, these data suggest that the protective association of T cell therapy against prostate cancer has become increasingly evident with the evolution of immunosuppressive regimens. Notably, while Cox multivariate models demonstrated significant risk reduction during 2005-2014, the 2015–2019 cohort showed no statistically significant association. This non-significance may reflect the relatively short median follow-up in this recent era, warranting extended surveillance for definitive assessment.

Interestingly, in terms of survival analysis, the 5-year and 10-year survival rates of prostate cancer are better than the tumor free group after transplantation. The possible reasons are as follows: Patients with malignant tumors after KT have more regular follow-up, and prostate cancer have lower invasiveness and less distant metastasis. Regular physical examination and early treatment make the prognosis of patients with prostate cancer better than the tumor free group. Furthermore, prostate cancer diagnosis inherently requires sufficient post-transplant survival time to be detected. This immortal time bias may artificially inflate observed survival rates in the cancer cohort.

It is general acknowledged in tumor immunology that the occurrence and progression of tumors is due to the decrease in the body’s immune surveillance ability, which leads to the immune escape of abnormally growing potential tumor cells (43, 44). The decline in immune function implies natural immune tolerance, which may benefit patients in reducing transplant kidney rejection. In addition, transplant patients with HLA mismatch ≥ 3 would normally use stronger immunosuppressive regimens after surgery, and thus the decrease in the patient’s immune function may increase the risk of tumor development after transplantation.

The multivariate subgroup analysis of HLA mismatch 0–6 and match 0–6 showed no statistical significance. HLA-A, HLA-B 2-mismatch were risk factors of prostate cancer after KT, while HLA-DR both 1-mismatch and 2-mismatch were risk factors of prostate cancer after KT, which can be explained as follows: Firstly, HLA-A, HLA-B 2-mismatch increased the risk of prostate cancer after transplantation, while 1-mismatch results were not statistically significant, indicating that an increase in mismatches in HLA-A and HLA-B could add to the risk of prostate cancer after transplantation. HLA-A and HLA-B belong to MHC class I antigens, and former research have shown that abnormal MHC-I expression and function regulation may be hijacked by tumor cells to evade immune surveillance, thereby promoting tumor progression and impairing the efficacy of cancer immunotherapy (45–47). In addition, in the subgroup analysis of HLA-DR, 1-mismatch and 2-mismatch were both risk factors of prostate cancer after KT, indicating the special role of HLA-DR in HLA matching during transplantation. Previous studies on KT have found a significant correlation between the number of HLA-DR mismatches and the incidence of non-Hodgkin’s lymphoma and hip fractures (48). For example, the research based on hematopoietic stem cell transplantation (HSCT) by Biernacki has found out that most recurrent HSCT recipients have 3- to 12- fold reduction in HLA class II gene expression (49). IFN-γ exposure can reverse class II downregulation, while HLA class I expression is not downregulated, possibly due to the specific effect of CD4^+^T cells on HLA class II antigens during the immune response process after transplantation. In KT, the role of HLA-DR still requires more in-depth basic research.

In this study, we observed that HLA mismatch ≥ 3 also increases the risk of renal carcinoma after KT [1.05(1.03~1.09)] (**Supplementary Table 1**, **Supplementary Figure 2**). Further subgroup analysis showed that the results of HLA-A, HLA-B, and HLA-DR groups were not statistically significant (**Supplementary Figure 3**, **Supplementary Figure 4**), which indicate that there are differences in the impact of HLA mismatch in different types of tumors after KT, and further research is required to verify this conclusion.

Our study still has certain limitations. Firstly, the study could not explain the differences in HLA match and mismatch between kidney transplant donors and recipients. As shown in **Table 2**, there is no corresponding relationship between HLA match and mismatch due to the presence of NA. Specifically, an NA cannot be classified as either a match or a mismatch. This ambiguity can lead to analytical inconsistencies. In the case of HLA 1-mismatch, 3-match, 4-match, and 5-match can be present. However, the Cox multivariate analysis and subgroup analysis results of this study showed a one-to-one correspondence between match and mismatch, which is inconsistent with the expected results. This issue has been mentioned before, but still remained unclear (50). The reason may be attributed to the advancement of HLA detection technology, which in other words, the HLA loci that could not be detected before 2000 can now be detected, resulting in very few cases of HLA typing as NA. In this case, the Cox multivariate analysis results of HLA match and mismatch are consistent. Secondly, this retrospective study lacks control interventions for all confounding factors. Although the variables collected in UNOS are sufficiently rich, they still do not fully cover all the baseline characteristics of patients, which is also one of the drawbacks of retrospective studies. Thirdly, our study observed higher survival rates in kidney transplant recipients diagnosed with prostate cancer compared to non-cancer controls. Specifically, both 5-year and 10-year survival outcomes were significantly better in the prostate cancer group. This counterintuitive finding may be attributable to immortal time bias, as patients must survive long enough after transplantation to receive the cancer diagnosis. This methodological limitation could potentially inflate survival estimates in the cancer group. The persistent influence of this bias cannot be fully excluded. Further validation studies using validated methods and extended follow-up analyses are warranted to confirm these findings.

**Table 2:** In the case of HLA 1-mismatch between recipient and donors, 5-match, 4-match, and 3-match can be present with donor A, B, and C, respectively.

HLA typing	Recipient	Donor A	Donor B	Donor C
HLA-A	26	26	26	26
	29	29	NA	22
HLA-B	7	6	7	7
	8	8	6	NA
HLA-DR	10	10	10	NA
	17	17	17	17
mismatch		1	1	1
match		5	4	3

## 5 Conclusions

This retrospective study quantitatively analyzed the impact of: (1) baseline patient characteristics; (2) HLA mismatch; and (3) HLA subtype mismatch on the incidence of prostate cancer after KT. The use of T cell therapy could reduce the risk of prostate cancer after transplantation. HLA mismatch ≥ 3 is a risk factor of prostate cancer after KT. HLA-A and HLA-B 2-mismatch are risk factors of prostate cancer after KT, while HLA-DR 1-mismatch and 2-mismatch are both risk factors of prostate cancer after KT. The research contributes to emphasize advantages of T cell therapy from the perspective of prostate cancer occurrence, focus on HLA mismatch in KT and provide guidance for reasonable selection of HLA typing of prostate cancer.

## Data Availability

The data analyzed in this study were sourced from a publicly available dataset, available at: https://optn.transplant.hrsa.gov/data/request-data/.
