# Supplementary material for: Analyzing the impact of human leukocyte antigen mismatch on the incidence of prostate cancer and the advantage of T cell therapy in patients after kidney transplantation based on the United Network for Organ Sharing database
Source: Front Oncol. 2025 Sep 10;15:1562869. doi: 10.3389/fonc.2025.1562869 (PMC12457105; doi:10.3389/fonc.2025.1562869)
Supplement: Supplementary file 1 [file DataSheet1.docx]

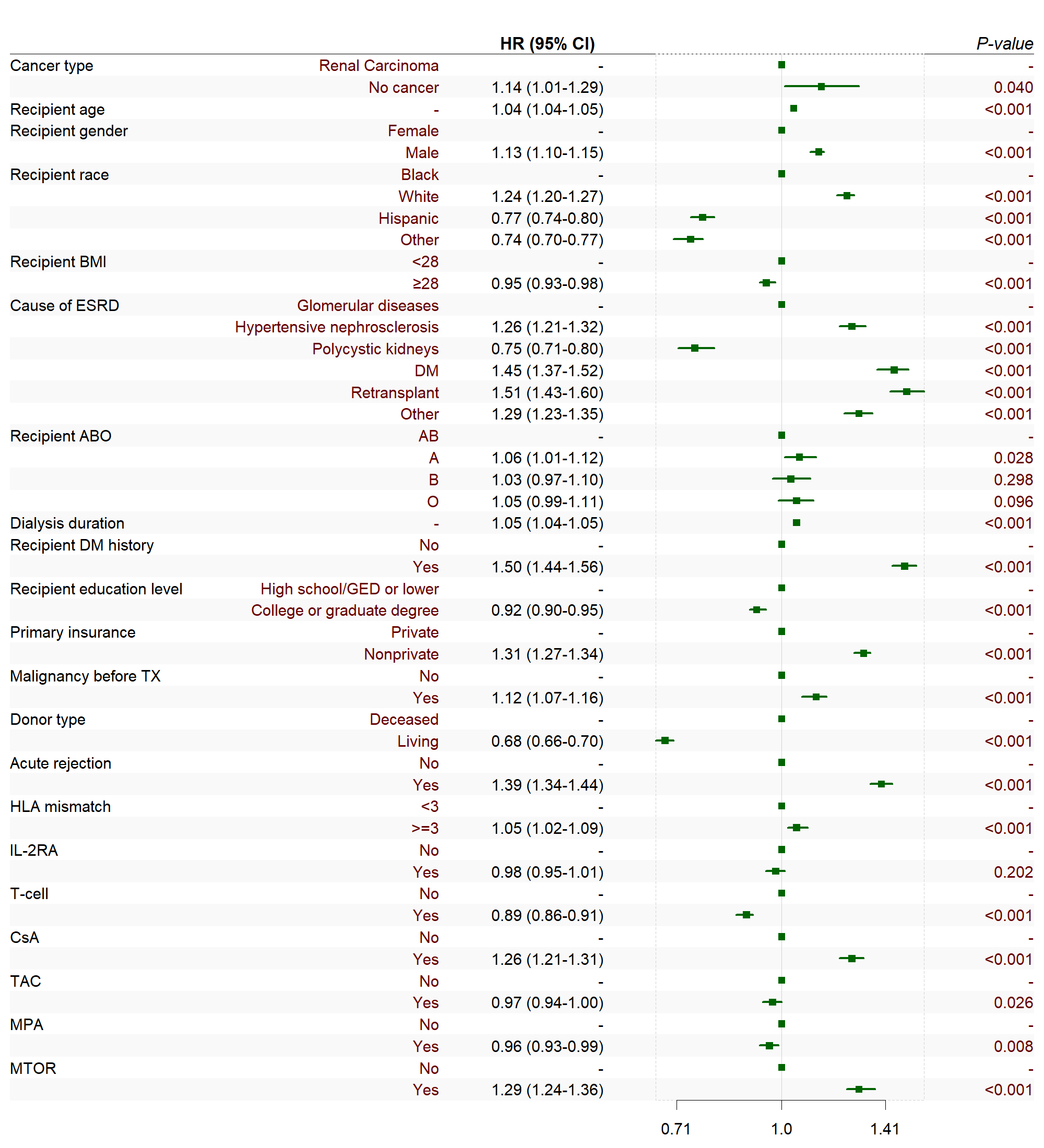


**Supplementary Figure 1** Cox multiple regression analysis of the influencing factors of renal carcinoma incidence after KT.
