# Supplementary material for: Analyzing the impact of human leukocyte antigen mismatch on the incidence of prostate cancer and the advantage of T cell therapy in patients after kidney transplantation based on the United Network for Organ Sharing database
Source: Front Oncol. 2025 Sep 10;15:1562869. doi: 10.3389/fonc.2025.1562869 (PMC12457105; doi:10.3389/fonc.2025.1562869)
Supplement: Supplementary file 2 [file DataSheet2.docx]

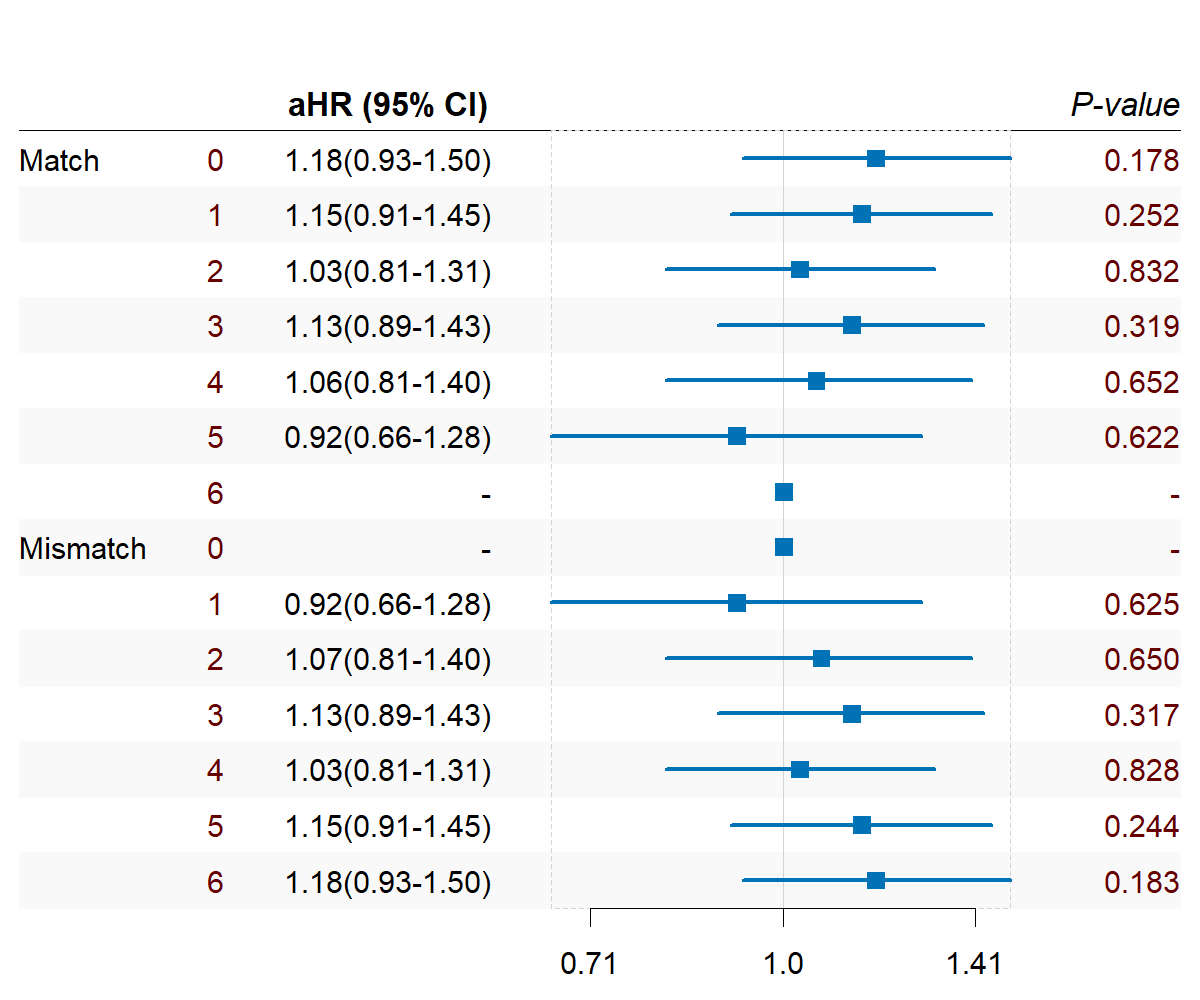


**Supplementary Figure 2** Multiple factor analysis of HLA match 0-6 and mismatch 0-6 of renal carcinoma incidence after KT.
