# Supplementary material for: Analyzing the impact of human leukocyte antigen mismatch on the incidence of prostate cancer and the advantage of T cell therapy in patients after kidney transplantation based on the United Network for Organ Sharing database
Source: Front Oncol. 2025 Sep 10;15:1562869. doi: 10.3389/fonc.2025.1562869 (PMC12457105; doi:10.3389/fonc.2025.1562869)
Supplement: Supplementary file 4 [file DataSheet4.docx]

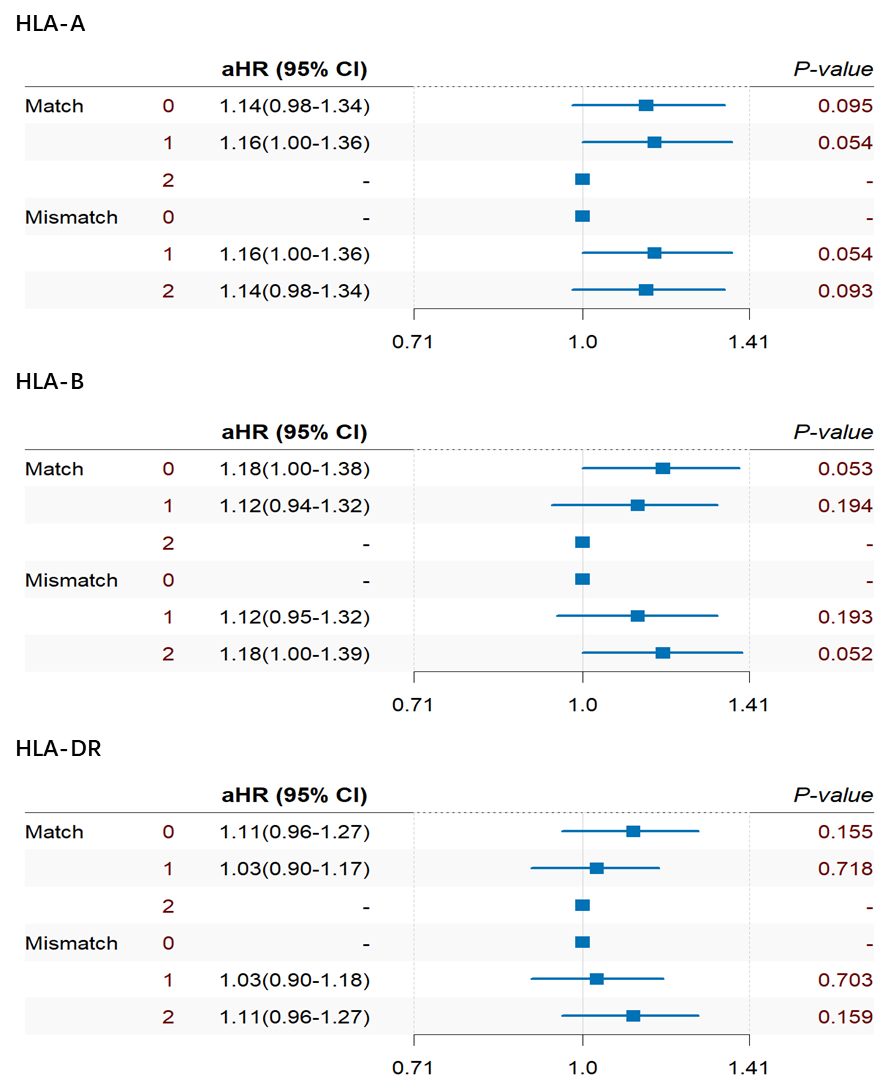


**Supplementary Figure 4** Cox multiple regression analysis of HLA-A, HLA-B, HLA-DR match 0-2 and mismatch 0-2 of renal carcinoma incidence after KT.
