# Supplementary material for: Analyzing the impact of human leukocyte antigen mismatch on the incidence of prostate cancer and the advantage of T cell therapy in patients after kidney transplantation based on the United Network for Organ Sharing database
Source: Front Oncol. 2025 Sep 10;15:1562869. doi: 10.3389/fonc.2025.1562869 (PMC12457105; doi:10.3389/fonc.2025.1562869)
Supplement: Supplementary file 5 [file Table1.docx]

**Supplementary Table 1** Analysis of influencing factors for the incidence of renal carcinoma in 268 994 recipients after KT.

|  |  |  |  |
| --- | --- | --- | --- |
|  | No cancer | Renal Carcinoma | p |
| n | 267084 | 2072 |  |
| Age (median [IQR]) | 51.00 [40.00, 60.00] | 54.00 [45.00, 62.00] | <0.001 |
| Gender = Male (%) | 160015 (59.9) | 1498 (72.3) | <0.001 |
| Recipient race (%) |  |  | <0.001 |
| White | 130915 (49.0) | 1047 (50.5) |  |
| African American | 71734 (26.9) | 692 (33.4) |  |
| Hispanic | 43813 (16.4) | 224 (10.8) |  |
| Asian | 15908 (6.0) | 75 ( 3.6) |  |
| Other | 4714 (1.8) | 34 (1.6) |  |
| BMI ≥28 (%) | 119026 (44.6) | 1051 (50.7) | <0.001 |
| Cause of ESRD (%) |  |  | <0.001 |
| Glomerular diseases | 49749 (18.6) | 463 (22.3) |  |
| Hypertensive nephrosclerosis | 59268 (22.2) | 600 (29.0) |  |
| Polycystic kidneys | 22974 (8.6) | 132 (6.4) |  |
| DM | 67230 (25.2) | 403 (19.4) |  |
| Retransplant | 20603 (7.7) | 149 (7.2) |  |
| Other | 47260 (17.7) | 325 (15.7) |  |
| Dialysis duration (median [IQR]) | 3.00 [1.00, 5.00] | 3.00 [1.00, 5.00] | 0.449 |
| Recipient education level (%) |  |  | 0.069 |
| High school/GED or lower | 116848 (43.7) | 865 (41.7) |  |
| College or graduate degree | 123788 (46.3) | 976 (47.1) |  |
| Unknown | 26448 (9.9) | 231 (11.1) |  |
| Private insurance (%) |  |  | 0.377 |
| Yes | 96852 (36.3) | 732 (35.3) |  |
| No | 170232 (63.7) | 1340 (64.7) |  |
| ABO_FACTOR (%) |  |  | 0.462 |
| A | 98669 (36.9) | 771 (37.2) |  |
| B | 34551 (12.9) | 245 (11.8) |  |
| AB | 13060 (4.9) | 98 (4.7) |  |
| O | 120804 (45.2) | 958 (46.2) |  |
| Living transplantation (%) | 602 (0.2) | 27 (1.3) | <0.001 |
| Acute rejection (%) | 97352 (36.5) | 719 (34.7) | 0.104 |
| HLA mismatch (%) |  |  | 0.86 |
| <3 | 54441 (20.4) | 414 (20.0) |  |
| ≥3 | 211770 (79.3) | 1652 (79.7) |  |
| Unknown | 873 (0.3) | 6 (0.3) |  |
| immunosuppression induction |  |  |  |
| Interleukin-2 receptor subunit alpha (%) | 68526 (25.7) | 565 (27.3) | 0.099 |
| T cell therapy (%) | 156955 (58.8) | 1130 (54.5) | <0.001 |
| immunosuppression maintenance |  |  |  |
| CSA (%) | 28246 (10.6) | 250 (12.1) | 0.031 |
| TAC (%) | 165338 (61.9) | 1477 (71.3) | <0.001 |
| MPA (%) | 196240 (73.5) | 1697 (81.9) | <0.001 |
| MTOR (%) | 15333 (5.7) | 94 ( 4.5) | 0.021 |
|  |  |  |  |
